# Influence of *KRAS* mutation subtypes on response to neoadjuvant chemoradiotherapy in locally advanced rectal cancer: meta-analysis

**DOI:** 10.1093/bjsopen/zrag080

**Published:** 2026-08-04

**Authors:** Iannish D Sadien, George Cooper, Veronica Phillips, Heman Joshi, James Wheeler, R Justin Davies

**Affiliations:** Cancer Research UK Cambridge Institute, Li Ka Shing Centre, Robinson Way, Cambridge, UK; Cambridge Colorectal Unit, Cambridge University Hospitals NHS Foundation Trust, Cambridge, UK; School of Clinical Medicine, University of Cambridge, Cambridge, UK; School of Clinical Medicine, University of Cambridge, Cambridge, UK; University of Cambridge Medical Library, University of Cambridge School of Clinical Medicine, Cambridge, UK; Cambridge Colorectal Unit, Cambridge University Hospitals NHS Foundation Trust, Cambridge, UK; Cambridge Colorectal Unit, Cambridge University Hospitals NHS Foundation Trust, Cambridge, UK; Cambridge Colorectal Unit, Cambridge University Hospitals NHS Foundation Trust, Cambridge, UK; School of Clinical Medicine, University of Cambridge, Cambridge, UK

**Keywords:** complete response, resistance, systematic review, transcriptomics, genetics, RAS

## Abstract

**Background:**

*KRAS* mutations are common in colorectal cancer, but the impact of *KRAS* mutation subtypes on treatment response remains poorly understood. This research aimed to investigate whether different *KRAS* mutations influence pathological complete response (pCR) rates after neoadjuvant chemoradiotherapy in locally advanced rectal cancer (LARC).

**Methods:**

A systematic review and meta-analysis of studies describing genetic determinants of response to neoadjuvant chemoradiotherapy in LARC was conducted, searching for manuscripts published up to March 2026. The primary outcome of interest was the odds ratio for *KRAS* mutations and pCR. A random-effects model estimated the pooled effect size of *KRAS* mutations within and outside exon 2 on pCR. Genomic data sets were analysed to investigate the molecular characteristics of *KRAS* exon 2 and non-exon 2 mutant rectal cancers and their impact on overall and disease-free survival. Finally, a transcriptomic data set was analysed to elucidate the underlying response mechanisms.

**Results:**

Out of 11 537 manuscripts identified, 15 studies (3354 patients) were included in the meta-analysis. The odds ratio for any *KRAS* mutation and pCR was 0.48 (95% confidence interval 0.32 to 0.70), indicating reduced odds of pCR in *KRAS*-mutant LARC. Subgroup analysis revealed that *KRAS* mutations in exon 2 accounted for this effect, whereas variants outside exon 2 had no influence (odds ratio 0.96, 95% confidence interval 0.11 to 8.49). Analysis confirmed poorer disease-free survival in patients with exon 2 alterations (*P* = 0.019) as well as poorer overall survival (*P* = 0.047). Transcriptomic analysis revealed that non-exon 2 *KRAS*-mutant tumours were enriched for inflammatory signalling pathways, suggesting that these tumours represent a subgroup with high immune infiltration.

**Conclusion:**

The presence of *KRAS* mutations adversely affects pCR odds after neoadjuvant chemoradiotherapy in LARC, but this effect is specific to exon 2 mutations.

## Teaser

Approximately 40% of rectal cancers harbour mutations in the *KRAS* oncogene. Here the authors show that the presence of these mutations adversely affects response to neoadjuvant therapy. Furthermore the particular subtype of alteration in *KRAS* also influences response and prognosis.

## Introduction

Colorectal cancer (CRC) represents a significant global health burden, ranking as the third most common malignancy worldwide^1^. Among these, locally advanced rectal cancer (LARC) presents distinct therapeutic challenges that necessitate a multidisciplinary approach. The standard of care for LARC typically involves neoadjuvant chemoradiotherapy (nCRT) followed by total mesorectal excision, which has been shown to improve local control and sphincter preservation rates compared with surgery alone^2–5^.

Despite standardized treatment protocols, response to nCRT varies considerably among patients with LARC, with pathological complete response (pCR) rates ranging from 10% to 30%^6^. This variability highlights the need for reliable biomarkers that can predict treatment response and guide therapeutic decision-making. Among the various molecular alterations in rectal cancer, mutations in the *KRAS* proto-oncogene have emerged as potential predictors of treatment response and survival outcomes.

KRAS is a member of the RAS family of membrane-anchored guanosine triphosphate GTP)/guanosine diphosphate-binding proteins that play a crucial role in intracellular signal transduction, particularly from the epidermal growth factor receptor (EGFR)^7^. In its active state, KRAS activates downstream effectors, including Raf, phosphatidylinositol 3-kinase, and Ral guanine nucleotide-dissociation stimulator, ultimately regulating cellular proliferation, survival, and differentiation^8^. Mutations in *KRAS*, occurring in approximately 35–45% of LARC cases, result in the persistent accumulation of active GTP-bound KRAS protein, leading to the constitutive activation of proproliferative signalling pathways.

The prognostic significance of *KRAS* alterations in CRC has been the subject of considerable debate. Although several studies have demonstrated that *KRAS* mutations confer resistance to anti-EGFR therapy in metastatic CRC, the prognostic value of these mutations in LARC treated with nCRT remains controversial^9^. Some studies suggest that *KRAS* mutations are associated with worse overall survival (OS) and an increased risk of relapse, whereas others report no significant association between *KRAS* status and treatment response or long-term outcomes^10–12^. These discrepancies may be attributed to variations in study methodologies, patient populations, sample sizes, and treatment modalities. Moreover, most studies have treated *KRAS* mutations as a homogeneous entity, without considering the potential differential impact of specific mutation subtypes. *KRAS* mutations most commonly occur in codons 12 and 13, with G12D, G12V, and G13D being the most prevalent variants^13^. Emerging evidence^14,15^ suggests that different *RAS* mutation subtypes may confer distinct biological behaviours and therapeutic implications.

A key endpoint in evaluating the efficacy of neoadjuvant treatment is the achievement of a pCR, defined as the absence of a residual tumour at histopathological assessment after surgery. Achieving a pCR has been associated with improved long-term outcomes in patients with rectal cancer^5^. Understanding the specific roles of different *KRAS* mutation subtypes in determining the response to nCRT in LARC could significantly enhance the ability to stratify patients for treatment and to develop personalized therapeutic strategies. This study aims to investigate the relationship between specific *KRAS* mutation subtypes and response to nCRT in patients with locally advanced rectal cancer, with particular focus on pathological response.

## Methods

A systematic review and meta-analysis were first conducted to isolate the effect of *KRAS* alterations in LARCs. Subsequently, a large, publicly available genomic data set was analysed to identify oncological outcomes between exon 2 and non-exon 2 *KRAS*-mutant rectal cancers. Finally, differential gene expression and gene set enrichment analyses were performed to identify potential underlying mechanisms for the observed effects.

### Systematic review and meta-analysis

A systematic review was conducted and reported in accordance with PRISMA guidelines^16^. MEDLINE (via Ovid), Embase (via Ovid), the Cochrane Library, Web of Science (Core Collection), and Scopus were searched from inception to March 2026 to identify studies reporting on genetic determinants of pathological response to neoadjuvant radiotherapy and/or chemotherapy in LARC. No restrictions were made regarding publication type. The search strategy was peer-reviewed using the Peer Review of Electronic Search Strategies (PRESS) checklist^17^ and evaluated against the PRISMA-S guidelines^18^. To account for variation between thesaurus terms/controlled vocabulary, the search syntax was tailored, and searches were performed separately for each database. Results were imported to Endnote 21 for deduplication, using the method outlined by Bramer *et al*^19^. Where this was not possible, deduplication was performed were manually. Grey literature was not searched. The full search strategies used in each database are included in the *supplementary methods*.

### Study selection

Studies reporting pCR rates following standard nCRT or total neoadjuvant therapy (TNT) stratified by *KRAS* mutational status, in adults aged > 18 years with LARC were eligible for inclusion. Definitions of LARC were not always explicitly given in studies and likely varied. Similarly, neoadjuvant treatment schedules varied across studies, but these were consistent within individual studies between the *KRAS*-mutant and *KRAS*-wildtype cohorts.

Single-arm studies, non-comparative observational studies, and studies reporting on clinical response rates only (either in the setting of watch-and-wait protocols or before resection) were excluded. Studies describing mutation status without genomic sequencing of tumour biopsies, for example, using exploratory techniques such as circulating tumour DNA or transcriptomic analyses, were excluded to preserve internal validity and clinical applicability. Systematic reviews, narrative reviews, case reports, letters, editorials, and animal studies were excluded. However, a bidirectional cascade search of relevant references was conducted to identify additional eligible studies. Two reviewers independently screened titles, abstracts, and full texts of potentially eligible studies. Any disputes were resolved by discussion. Screening was carried out using the Rayyan web app (http://rayyan.qcri.org/)^20^.

### Data extraction

Two reviewers independently extracted and tabulated prespecified data from included studies, including study characteristics (citation, study design, setting, location, number of participants, method of DNA sequencing (including next generation (NGS), Sanger, and capillary electrophoresis sequencing), exons assayed, overall pCR rate and OS, and applicable inclusion and exclusion criteria), nCRT protocol, and reported outcome measures. The S:CORT (Stratification in COloRectal Tumours)^21^ data set was selected as it represents one of the largest prospectively collected, multiomic CRC cohorts with paired clinical and transcriptomic data publicly available. The Grampian cohort was used specifically as it includes RNA microarray data with associated consensus molecular subtype (CMS) classification and cell type deconvolution data, enabling comprehensive transcriptomic characterization of *KRAS* mutant subgroups. No additional search or restriction criteria were applied beyond the availability of *KRAS* mutational status and RNA microarray data.

### Outcomes of interest

The primary outcome was pCR rate as it is an objective measure of the biological efficacy of neoadjuvant therapy. Studies that did not report pCR were not included. A prespecified sensitivity analysis was performed to investigate the association between the nCRT regime and pCR. OS and disease-free survival (DFS) were evaluated based on *KRAS* mutational status. Finally transcriptomic differences were evaluated using gene set enrichment analysis using Hallmark Pathways, CMS, and cell type deconvolution.

### Quality assessment

Study quality was assessed by two independent reviewers (I.D.S. and G.M.C.) using the Newcastle–Ottawa Scale for non-randomized studies (http://www.ohri.ca/programs/clinical_epidemiology/oxford.htm).

### Survival analyses

OS and DFS analyses were performed using the Kaplan–Meier method using the *survival* and *survminer* R packages^22,23^. Only patients with *KRAS* mutational data were included for this analysis. Definitions and source data have previously been published and made publicly available^24^.

### Transcriptomic comparison

Differential gene expression analysis was performed on RNA microarray data from the Grampian cohort of the S:CORT data set using *limma*^25^. Methods for sample collection, processing, RNA microarray sequencing, CMS, and cell type deconvolution have previously been published^21^. Gene set enrichment analysis was performed based on the Hallmark Pathways using the *camera* package^26^. Comparison of CMSs and cell type deconvolution for immune cell enrichment analyses were performed on data already generated by the S:CORT study.

### Statistics

Data from included studies were pooled for meta-analysis using the *meta* package^27^. Analysis of unadjusted dichotomous variables was performed, and odds ratios (OR) were reported as the event rate (pCR) was judged to be relatively rare in some studies. A random-effects model was used due to heterogeneity in treatment regimens in the included studies. A *P* value of less than 0.05 was considered statistically significant. Interstudy heterogeneity was assessed using the *I*^2^ statistic, and reporting bias was assessed using funnel plots when more than 10 studies reported the same outcome. *Post hoc* sensitivity analyses were performed to assess the impact of studies with substantial influence on the effect size or outcome heterogeneity using the *dmetar* package (https://github.com/MathiasHarrer/dmetar). Missing data were excluded from analyses. Imputation was not attempted. All analyses were performed using *R* (version 4.4.2 2024–10–31).

## Results

**Fig. 1:**
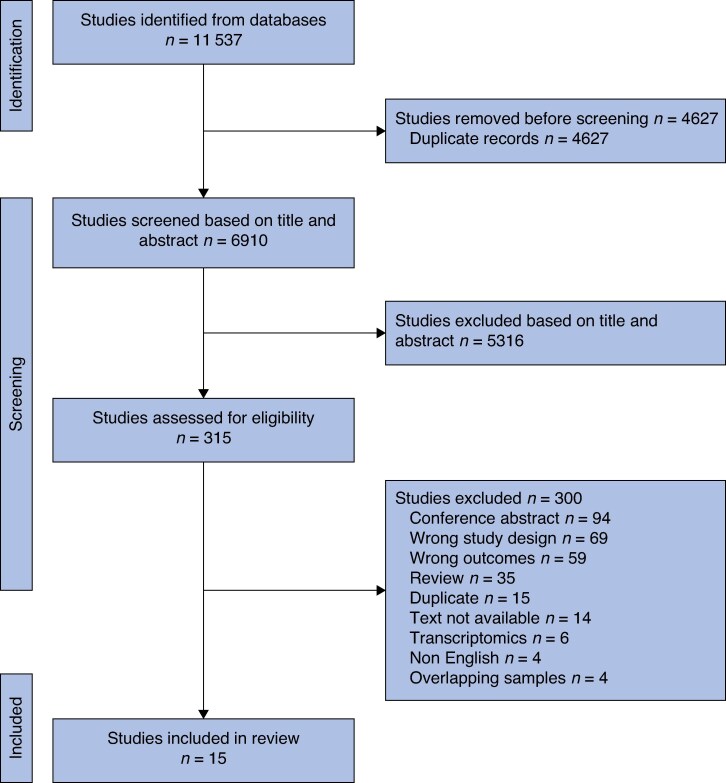
PRISMA flow diagram showing literature search results

A total of 11 537 publications were obtained from the search, and 315 full-text articles were assessed. Of these, 15 studies comprising 3354 patients were included^15,28–44^ (*Fig. 1*).

### *KRAS* mutations and pCR rates

**Fig. 2:**
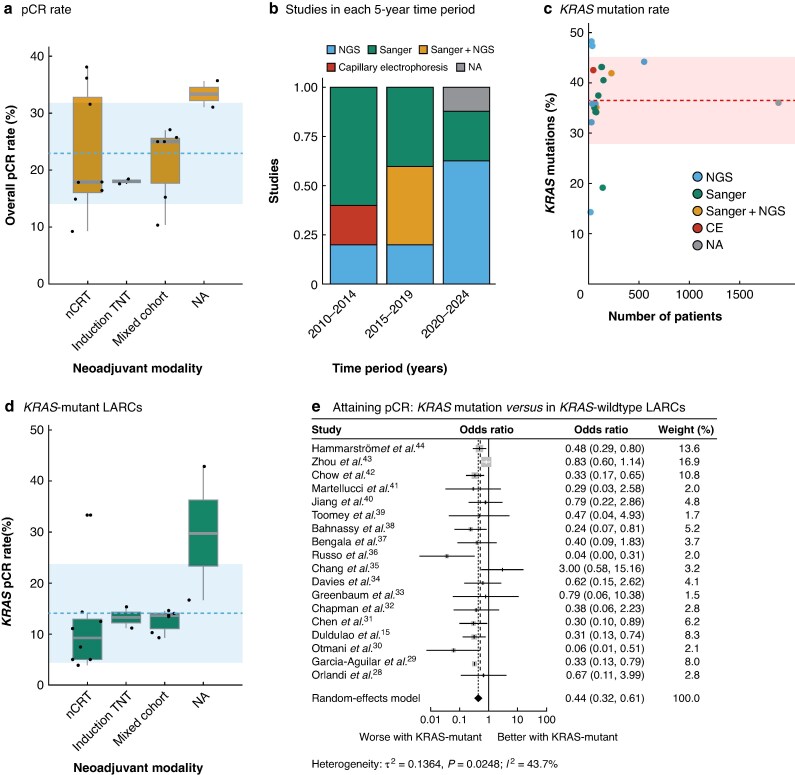
*KRAS* mutations negatively impact pCR rates **a** Box plot of reported pCR rate in the 15 included studies by neoadjuvant treatment modality. Dashed line represents the mean pCR across the 15 studies with band denoting one standard deviation around the mean. **b** Stacked bar chart showing proportion of studies in each 5-year time period using particular DNA sequencing methods. **c** Scatter plot of *KRAS* mutation frequency (percentage) *versus* the sample size of individual studies. Each dot represents an individual study, coloured by the method of DNA sequencing employed. Dashed line represents mean *KRAS* mutation frequency with band denoting one standard deviation around the mean. *P* = 0.440. **d** Box plot pCR rate for *KRAS*-mutant LARCs only stratified by neoadjuvant treatment modality. Blue dashed line represents the mean pCR across the 15 studies with blue band denoting one standard deviation around the mean. *P* = 0.250. **e.** Forest plot showing the odds ratio of attaining pCR in the presence of a *KRAS* mutation *versus* in *KRAS*-wildtype LARCs. Figures in brackets represent 95% confidence intervals. pCR, pathological complete response rate; LARC, locally advanced rectal cancer; nCRT: neoadjuvant chemoradiotherapy; TNT: total neoadjuvant therapy; NA, not available (not reported in the study); NGS, next generation sequencing; CE, capillary electrophoresis.

Full study characteristics are presented in *Table S1*. Eight studies used nCRT, two used TNT, two did not report the neoadjuvant therapy modality, and the remaining studies had mixed cohorts (nCRT and TNT). The overall mean pCR rate across the 15 studies was 14.5% (*Fig. 2a*). There was no statistically significant difference in pCR rates with the different neoadjuvant modalities in the included studies (*Fig. 2a*). The use of NGS became more prevalent with time, likely reflecting increasing ease of access and decreasing costs (*Fig. 2b*). Of all LARCs in the included studies, 37.1% harboured *KRAS mutations.* The frequency of *KRAS* alterations was not influenced by the DNA sequencing method or study sample size (*Fig. 2c*). Considering *KRAS*-mutant LARCs alone, the mean pCR rate was 10.4% and was not dependent on the neoadjuvant treatment modality (*Fig. 2d*). To determine the effect of *KRAS* mutation status on pCR rate after neoadjuvant treatment, a meta-analysis was performed. In accordance with previous reports, this revealed that the presence of a *KRAS* alteration leads to significantly lower rates of pCR compared with *KRAS*-wildtype LARCs (OR 0.48, 95% confidence interval (c.i.) 0.32 to 0.70) (*Fig. 2e*). The 15 included studies showed low-to-moderate between-study heterogeneity (*I^2^* = 45.6%). Influence analysis revealed that the study by Zhou *et al*.^43^ was highly influential and contributed significantly to heterogeneity (*Fig. S1* and *Fig. S2*). A sensitivity analysis was performed by omitting this study. This demonstrated a conserved effect size (OR 0.42, 95% c.i. 0.31 to 0.58) with low between-study heterogeneity (*I^2^* = 23.5%) (*Fig. S3*). In summary, these data suggest that the presence of a *KRAS* mutation strongly predicts reduced odds of pCR after neoadjuvant therapy in LARC.

### Exon 2 mutations and resistance to neoadjuvant therapy

**Fig. 3:**
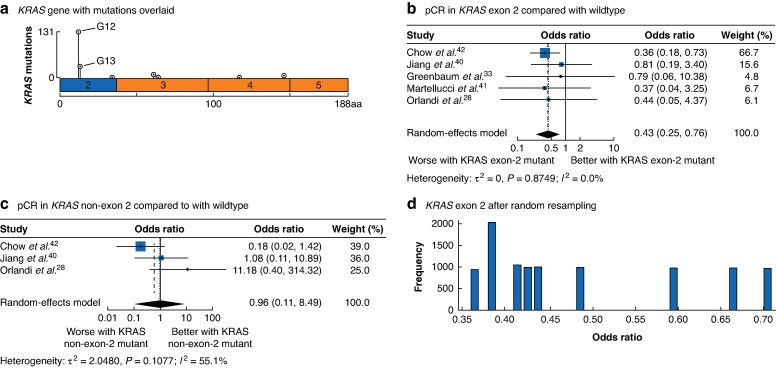
Negative impact of *KRAS* mutation on pCR is limited to exon 2 alterations **a** Schematic of coding region of the *KRAS* gene with frequency of mutations in different regions overlaid. Exon 2 coloured in blue with other exons in yellow. **b** Forest plot showing odds ratios of pCR in *KRAS* exon 2 mutant samples compared to wildtype samples. **c** Forest plot showing odds ratios of pCR in *KRAS* non-exon 2 mutant samples compared to wildtype samples. **d** Histogram of distribution of odds ratios for *KRAS* exon 2 mutant samples after random resampling (10 000 resamples).

Next, to investigate the influence of different *KRAS* mutational subtypes on pathological response rates, the individual mutant codon positions in the 15 included studies were tallied. Of the studies that reported the exact locations of *KRAS* mutations, most variants were in exon 2 of the gene, specifically at codons 12, 13, and 34 (*Fig. 3a*). The remaining variants were scattered throughout the gene’s coding region (hereafter termed non-exon 2 mutations). A meta-analysis was performed isolating the influence of a *KRAS* exon 2 mutation on pCR. This analysis confirmed significantly lower odds of achieving pCR in this setting (OR 0.43, 95% c.i. 0.25 to 0.76) (*Fig. 3b*). Interestingly, the between-study heterogeneity in this analysis was very low (*I^2^* = 0%), suggesting that a mutation subtype was responsible for a significant amount of heterogeneity seen in the analysis, considering all *KRAS* mutations together. This meta-analysis was repeated for *KRAS* non-exon 2 mutations, revealing a non-significant OR (0.96, 95% c.i. 0.11 to 8.49) (*Fig. 3c*). To rule out that the significant difference between exon 2 and non-exon 2 *KRAS* alterations was due to sample size differences between the two groups, random subsampling of the group of studies reporting on exon 2 was performed. The distribution of ORs derived from this analysis was non-overlapping with that observed in the non-exon 2 cohort, suggesting that sample size differences were not a contributory factor and that the difference in pCR rates was real (*Fig. 3d*).

### Exon 2 mutations and survival

**Fig. 4:**
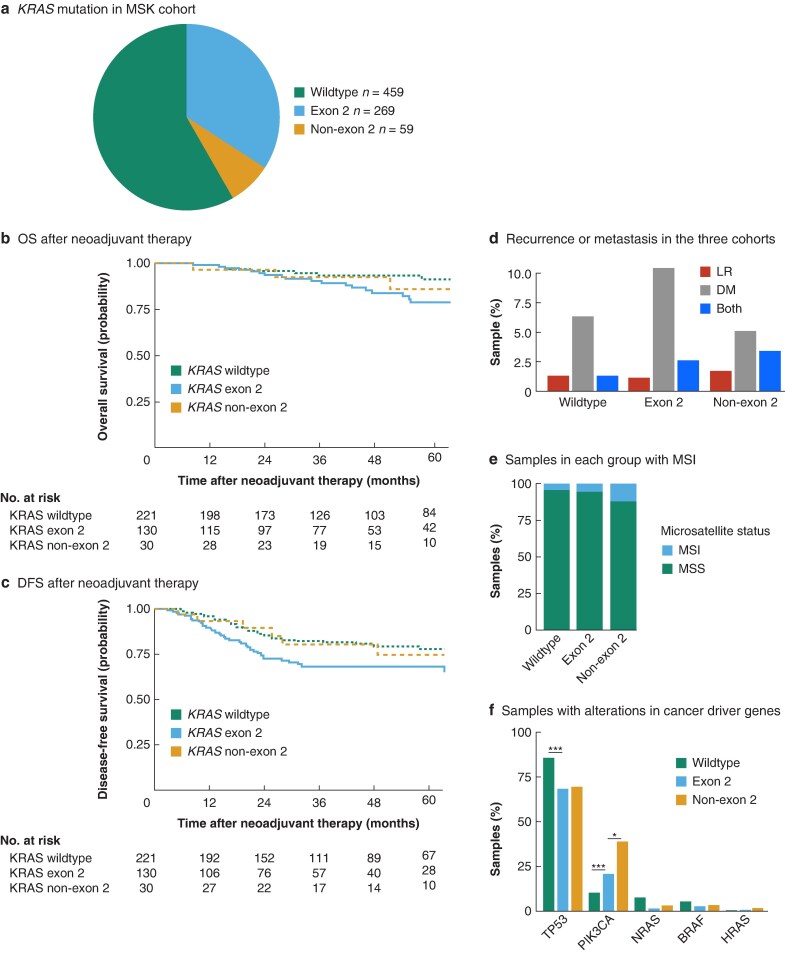
*KRAS* exon 2 confers worse survival in rectal cancer **a** Pie chart of *KRAS* mutation status of the 787 rectal cancer samples in the Memorial Sloan-Kettering (MSK) cohort. **b** Kaplan–Meier curve of OS following neoadjuvant therapy depending on *KRAS* mutation status. *P* = 0.047. **c** Kaplan–Meier curve of DFS following neoadjuvant therapy comparing *KRAS* wildtype, *KRAS* exon 2, and *KRAS* non-exon 2 rectal cancers. *P* = 0.019. **d** Bar plot of patterns of recurrence or metastasis in the three cohorts. **e** Stacked bar chart showing proportion of samples in each group with microsatellite instability. *P* = 0.056. **f** Bar chart showing percentage of samples in each group with alterations detected in selected cancer driver genes. OS, overall survival; DFS, disease-free survival; LR, local recurrence only; DM, distant metastasis only; MSI, microsatellite instability; MSS, microsatellite stable. *P < 0.05; **P < 0.01; ***P < 0.001.

Having established that *KRAS* mutational subtypes influence pCR rates in LARCs, the authors next sought to investigate their impact on survival more generally in rectal cancer. To do this, a previously published publicly available genomic data set^24^ was analysed. This resource comprises curated clinical and genetic sequencing data from 787 patients with rectal cancer. Of these, 328 of 787 (about 40%) had *KRAS* mutations, with most (82%) occurring in exon 2 (*Fig. 4a*). The presence of exon 2 alterations was associated with significantly reduced OS odds following neoadjuvant therapy compared with *KRAS* wildtype tumours (log-rank *P* = 0.047) (*Fig. 4b*). Additionally, there were significant differences in DFS with *KRAS* exon 2 mutations conferring poorer DFS (log-rank *P* = 0.019). Importantly, there were no differences between *KRAS* wildtype and non-exon 2 *KRAS* mutant cancers, suggesting that the latter behave more like wildtype than exon 2 mutants (*Fig. 4c*). Most disease relapses occurred in the form of distant metastases in all groups, with that difference in proportions being more pronounced in exon 2 mutant samples (*Fig. 4d*). The described differences in OS and DFS between exon 2 and non-exon 2 were not explained by differences in patient characteristics or tumour staging (*Table S2*), or microsatellite status (*Fig. 4e*). Exon 2 and non-exon 2 samples were also similar in terms of mutations in other cancer driver genes, with an enrichment in *PIK3CA* alterations in non-exon 2 samples (*Fig. 4f*).

### Transcriptomic profiling of *KRAS*-mutant cancers

**Fig. 5:**
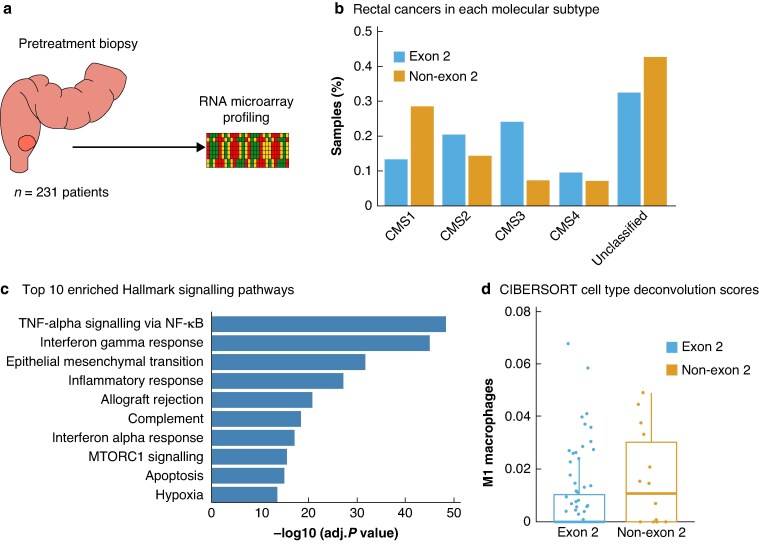
*KRAS* non-exon 2 samples are more immune-activated **a** Schematic of experimental protocol for RNA microarray analysis from pretreatment biopsies of rectal cancer. **b** Grouped bar plot showing proportion of rectal cancers in each CMS group. **c** Bar plot showing top 10 differentially expressed Hallmark Pathways from gene set enrichment analysis comparing *KRAS* non-exon 2 mutant cancers to exon 2 mutant samples. **d** Box plot showing CIBERSORT cell type deconvolution scores for M1-polarized macrophages between exon 2 and non-exon *KRAS* mutant samples. *P* = 0.048. There are 83 cancers in exon 2 and 14 cancers in non-exon groups. CMS, consensus molecular subtype; TNF, tumour necrosis factor; NF-κB, nuclear factor kappa B; MTORC1, mechanistic target of rapamycin complex 1; adj., adjusted.

To understand the molecular basis for the difference in phenotypes between exon 2 and non-exon 2 *KRAS* mutant cancers, transcriptomic data from the S:CORT study were analysed comparing the RNA microarray profiles of these two groups (85 exon 2, 14 non-exon 2) (*Fig. 5a*). No single gene crossed the significance threshold for differential gene expression between the two groups (*Fig. S4a*). Utilizing the well-established CMS to cluster these samples transcriptionally, the authors showed that most classified *KRAS* exon 2 mutant cancers were of the CMS3 group (enriched in *KRAS* mutations and metabolically dysregulated) as expected^45^ (*Fig. 5b*). Conversely, classified non-exon 2 mutant samples were depleted in CMS3 and instead showed enrichment of the CMS1 subtype (immune-infiltrated). Gene set enrichment analysis, looking at the Hallmark Pathways, confirmed the activation of immune-related pathways in non-exon 2 samples relative to exon 2 *KRAS* mutant cancers, with tumour necrosis factor (TNF)-alpha signalling, interferon gamma, and interferon alpha in particular being differentially activated (*Fig. 5c* and *Fig. S4a*). To identify potential differences in immune cell composition that may explain these observations, cell type deconvolution was performed using the transcriptomic data. Although no differences in regulatory T cells or cytotoxic CD8 or natural killer cells were found (*Fig. S4b–d*), this analysis suggested a trend towards a higher proportion of M1-polarized macrophages in non-exon 2 rectal cancers compared with exon 2 tumours (*Fig. 5d*). Importantly, M1 macrophage polarization has previously been implicated in mediating response to radiotherapy in solid cancers, suggesting this as the potential mechanism explaining the higher response rates seen in non-exon 2 *KRAS* mutant cancers^46,47^.

## Discussion

This study provides compelling evidence that *KRAS* mutation subtypes differentially impact neoadjuvant treatment response in locally advanced rectal cancer. Although the presence of a *KRAS* oncogene alteration significantly reduces the odds of achieving a pCR following neoadjuvant therapy, this effect is driven predominantly by mutations in exon 2, with non-exon 2 mutations showing no significant association with treatment resistance. The data strongly suggest that *KRAS* exon 2 mutations confer a more aggressive phenotype on rectal cancers, whereas non-exon 2 mutations behave more akin to *KRAS* wild-type cancers. This represents a paradigm shift in the understanding of *KRAS* as a predictive biomarker in rectal cancer. Previous studies that treated *KRAS* mutations as a homogeneous entity may have underestimated the true clinical significance of heterogeneity across mutation subtypes. These data suggest that exon 2 mutations, which comprise approximately 80% of all *KRAS* alterations in rectal cancer, are the primary drivers of treatment resistance, whereas the remaining 20% of mutations located elsewhere in the gene behave more similarly to wildtype tumours.

The biological basis for this differential behaviour is suggested by the transcriptomic analysis of pretreatment biopsies. Non-exon 2 mutant tumours demonstrate significantly higher immune activation, characterized by enrichment of interferon signalling pathways and increased M1-polarized macrophage infiltration. Importantly, therapeutic response to radiotherapy has been attributed to enhanced antigen presentation and cytotoxic T cell activation via M1-macrophage polarization^46^. Conversely, exon 2 mutant tumours predominantly exhibit the CMS3 transcriptional profile, characterized by metabolic dysregulation and relative immune exclusion, potentially contributing to their treatment-refractory phenotype. The differential response observed between *KRAS* subtypes may be explained by the influence of oncogenic *KRAS* signalling on the tumour immune microenvironment. For example, oncogenic KRAS can suppress interferon regulatory factor 2 (IRF2), a key regulator of immune signalling^48^. The loss of IRF2 results in the recruitment of myeloid-derived suppressor cells via the CXCL3–CXCR2 axis, creating a ‘cold’ immune-suppressive landscape that is inherently resistant to the proimmunogenic effects of radiotherapy. This aligns with the authors’ observation that exon 2 mutations are associated with a lack of immune-related gene set enrichment compared with their non-exon 2 counterparts, which maintain higher levels of interferon and TNF-α signalling. In this context, *KRAS* exon 2 status may be viewed as a surrogate marker for an immune-depleted, CMS3-like microenvironment. The authors’ findings that non-exon 2 *KRAS* mutants, which maintain an immune-enriched transcriptomic profile, retain sensitivity to nCRT further support the idea that the tumour microenvironment landscape, rather than the presence of a KRAS oncogene *per se*, dictates the clinical outcome.

These findings have some translational implications for clinical practice. Currently, *KRAS* mutational status is not routinely used to guide neoadjuvant treatment decisions in rectal cancer, partly due to conflicting reports regarding its prognostic value. The authors’ data suggest that a more nuanced approach, incorporating mutation subtype information, could significantly enhance treatment stratification. Patients harbouring exon 2 mutations may benefit from treatment intensification strategies. On the other hand, it remains to be determined whether non-exon 2 cancers in the metastatic setting would respond to anti-EGFR therapy, which is not currently recommended for *RAS*-mutant cancers^49^. Whereas these findings provide a foundation for more precise treatment stratification, the authors acknowledge that *KRAS* status alone cannot yet be used to dictate the omission of therapy. Instead, these results should guide the design of prospective randomized trials investigating whether patients with the *KRAS* exon 2-mutant benefit from intensified neoadjuvant regimens or novel radiosensitizers, while potentially exploring de-escalation for those with more favourable molecular profiles.

It is important to acknowledge that non-exon 2 *KRAS* mutations remain *bona fide* oncogenic drivers, as evidenced by their inclusion in comprehensive cancer gene databases and positive selection signatures in tumour evolution studies^50^. The paradoxical observation that these oncogenic mutations are associated with better treatment outcomes suggests that their role in dictating therapeutic response operates through mechanisms independent of their tumour-initiating properties. This phenomenon has been described in other cancer contexts, where driver mutations can paradoxically create therapeutic vulnerabilities through oncogene addiction or enhanced immunogenicity^51^.

Some limitations of this study warrant consideration. The retrospective nature of the included studies and variations in treatment protocols, although consistent within individual studies, may introduce heterogeneity. However, the low between-study heterogeneity observed in the exon 2-specific analysis suggests that mutation subtype is a robust predictor that transcends protocol variations. Additionally, the relatively small number of non-exon 2 mutations limits the statistical power of subgroup analyses, although the random resampling approach suggests that the observed differences are not merely due to sample-size effects. Whereas transcriptomic signatures point towards an immune-activated microenvironment in non-exon 2 mutants, the authors acknowledge the limitations of cell deconvolution in small cohorts. Furthermore, it is worth noting that clinical practice has evolved during the study period (2010–2024), with a move towards TNT and longer intervals to surgery, both of which are associated with higher absolute pCR rates^52^. Although the authors’ analysis showed no significant difference in the effect size of *KRAS* mutations between nCRT and TNT cohorts, the rising baseline pCR rate in modern practice should be considered when interpreting historical data.

The exact molecular mechanisms underlying the differential behaviour of *KRAS* mutation subtypes remain incompletely understood. Whereas this transcriptomic analysis provides insights into differences in the immune microenvironment, future studies should investigate whether specific exon 2 mutations exhibit differential binding affinities for downstream effectors or influence protein stability and localization differently from non-exon 2 variants. Understanding these mechanistic differences could inform the development of mutation-specific therapeutic strategies.

Looking forward, these findings suggest several avenues for clinical translation and further research. Prospective validation of the *KRAS* mutation subtype as a predictive biomarker in randomized clinical trials would provide definitive evidence for its clinical utility. Additionally, investigation of whether non-exon 2 mutant tumours are particularly responsive to immunomodulatory approaches, given their immune-activated phenotype, could identify novel therapeutic opportunities for this subset of patients.

In conclusion, this study demonstrates that the impact of *KRAS* mutations on neoadjuvant treatment response in LARC is highly dependent on mutation subtype. Exon 2 mutations confer significant treatment resistance and worse survival outcomes, whereas non-exon 2 mutations behave more similarly to wild-type tumours. These findings challenge the current paradigm of treating *KRAS* mutations as a uniform entity and provide a foundation for more precise, mutation subtype-guided therapeutic strategies in rectal cancer management. As personalized medicine continues to evolve, the incorporation of *KRAS* mutation subtype information into clinical decision-making algorithms represents a tangible step towards optimizing outcomes for patients with locally advanced rectal cancer.

## Supplementary Material

zrag080_Supplementary_Data

## Data Availability

Extracted data from studies included in the meta-analysis has been made publicly available for download (https://github.com/sadieni/kras_exon_rectal_cancer). Data from the S:CORT multiomic data set are available upon request to the S:CORT data access committee. No custom script was written for this study.
