# Supplementary material for: Influence of KRAS mutation subtypes on response to neoadjuvant chemoradiotherapy in locally advanced rectal cancer: meta-analysis
Source: BJS Open. 2026 Aug 4;10(4):zrag080. doi: 10.1093/bjsopen/zrag080 (PMC13436823; doi:10.1093/bjsopen/zrag080)
Supplement: Supplementary file 1 [file zrag080_supplementary_data.docx]

***KRAS* mutation subtypes influence response to neoadjuvant chemoradiotherapy in locally advanced rectal cancer**

Iannish D Sadien^1,2,3^, George Cooper^3^, Veronica Phillips^4^, Heman Joshi^2^, James Wheeler^2^, R Justin Davies^2,3^

^1^Cancer Research UK Cambridge Institute, Li Ka Shing Centre, Robinson Way, Cambridge, CB2 0RE, UK.

^2^Cambridge Colorectal Unit, Cambridge University Hospitals NHS Foundation Trust, Cambridge, CB2 0QQ, UK.

^3^University of Cambridge, Cambridge, CB2 0SP, UK.

^4^University of Cambridge Medical Library, University of Cambridge School of Clinical Medicine, Cambridge, CB2 0SP, UK.

**Corresponding author:**

Iannish Sadien, MA MB BChir MRCS PhD

**Address:** Cancer Research UK Cambridge Institute, Li Ka Shing Building, Robinson Way, Cambridge, CB2 0RE

**Email:** ids28@cam.ac.uk

**ORCID:** 0000-0002-3367-1252

**Supplementary Materials - Index**

| **Supplementary Methods** |  |
| --- | --- |
| Search strategy | *page 3-9* |
| **Supplementary Figures and Tables** |  |
| Table S1 – Characteristics of included studies | *page 10* |
| Figure S1 – Heterogeneity analysis | *Page 11* |
| Figure S2 – Influence analysis metrics | *Page 12* |
| Figure S3 – Forest plot of odds ratios after exclusion of influential study | *Page 13* |
| Table S2 – Baseline characteristics for patients in survival analysis | *Page 14* |
| Figure S4 – Transcriptomic analysis and cell type deconvolution | *Page 15* |

**Search strategy:**

The databases Medline (via Ovid), Embase (via Ovid), Cochrane Library, Web of Science (Core Collection), and Scopus were searched from inception to July 2024 by VP. The search strategy was peer-reviewed by two librarian colleagues of VP using the Peer Review of Electronic Search Strategies (PRESS) checklist (McGowan et al., 2016), and evaluated against the PRISMA-S guidelines (Rethlefsen et al., 2021). Databases were searched by VP separately, rather than multiple databases being searched on the same platform. The search syntax was adapted for each database, and to account for variation between thesaurus terms/controlled vocabulary across each database. Results were imported Rayyan by GMC for manual deduplication. The searches were rerun prior to submission in March 2026 to include any papers published between the initial searching, and submission for publication. Dates when searches were run are indicated in the results table. The full search strategies used in each database are documented below.

**Ovid MEDLINE(R) and Epub Ahead of Print, In-Process, In-Data-Review & Other Non-Indexed Citations, Daily and Versions <1946 to July 03, 2024>**

1	((Rect* or anus or anal) adj2 (cancer* or tumo* or neoplasm* or malignan* or oncolog*)).ti,ab,kw,kf.	43589

2	exp Rectal Neoplasms/	56096

3	1 or 2	69610

4	(KRAS or EGFR or APC or NTRK2 or BRAF or "B-raf" or "Kirsten rat sarcoma*" or "adenomatous polyposis coli" or "ptk receptor*" or "tyrosine kinase receptor*" or "epidermal growth factor receptor*" or "neurotrophic tyrosine receptor kinase*" or "transcriptomic signature*" or "RNA-based signature*" or "mismatch repair deficiency" or "microsatellite instability" or "high mutational burden*" or "genetic mutat*" or "cancer driver genes" or genomic*).ti,ab,kw,kf.	587164

5	exp Proto-Oncogene Proteins B-raf/	12511

6	exp Adenomatous Polyposis Coli/	6911

7	ErbB Receptors/	49126

8	exp Receptor Protein-Tyrosine Kinases/	172420

9	Microsatellite Instability/	5108

10	DNA Copy Number Variations/	13751

11	(("copy number" adj (variant* or change* or polymorph*)) or "ErbB Receptor*").ti,ab,kw,kf.	9068

12	4 or 5 or 6 or 7 or 8 or 9 or 10 or 11	720942

13	(regress* or respon* or recur* or surviv* or "disease free" or metastas* or mortal* or death or (treat* adj3 (outcome or effectiv* or efficac*))).ti,ab,kw,kf.	8581214

14	neoplasm recurrence, local/ or neoplasm regression, spontaneous/	152730

15	Survival/ or treatment outcome/	1200500

16	Disease-Free Survival/	82524

17	Neoplasm Metastasis/	114683

18	Mortality/	50082

19	Death/	20844

20	13 or 14 or 15 or 16 or 17 or 18 or 19	9284676

21	3 and 12 and 20	1499

**Embase <1974 to 2024 July 03>**

1	((Rect* or anus or anal) adj2 (cancer* or tumo* or neoplasm* or malignan* or oncolog*)).ti,ab,kw,kf.	65108

2	exp rectum cancer/	60806

3	1 or 2	85657

4	(KRAS or EGFR or APC or NTRK2 or BRAF or "B-raf" or "Kirsten rat sarcoma*" or "adenomatous polyposis coli" or "ptk receptor*" or "tyrosine kinase receptor*" or "epidermal growth factor receptor*" or "neurotrophic tyrosine receptor kinase*" or "transcriptomic signature*" or "RNA-based signature*" or "mismatch repair deficiency" or "microsatellite instability" or "high mutational burden*" or "genetic mutat*" or "cancer driver genes" or genomic* or "ErbB Receptor*").ti,ab,kw,kf.	824070

5	K ras protein/	31283

6	epidermal growth factor receptor/ or EGFR signaling/	111210

7	APC protein/	8457

8	brain derived neurotrophic factor receptor/	7840

9	protein tyrosine kinase/	67631

10	mismatch repair/	20000

11	microsatellite instability/	22876

12	gene mutation/	575857

13	genomics/	93336

14	("copy number" adj (variant* or change* or polymorph*)).ti,ab,kw,kf.	12745

15	4 or 5 or 6 or 7 or 8 or 9 or 10 or 11 or 12 or 13 or 14	1414586

16	(regress* or respon* or recur* or surviv* or "disease free" or metastas* or mortal* or death or (treat* adj3 (outcome or effectiv* or efficac*))).ti,ab,kw,kf.	11439016

17	cancer regression/	40713

18	cancer recurrence/	279883

19	progression free survival/ or survival/	512562

20	disease free survival/	116792

21	metastasis free survival/ or metastasis/	353937

22	mortality/	939146

23	death/	294190

24	treatment outcome/	1003927

25	16 or 17 or 18 or 19 or 20 or 21 or 22 or 23 or 24	12360863

26	3 and 15 and 25	3543

**Cochrane Library**

**Search Name:**

**Date Run:**	**04/07/2024 12:55:03**

**Comment:**

**ID**	**Search**	**Hits**

#1	(((Rect* or anus or anal) Near/2 (cancer* or tumo* or neoplasm* or malignan* or oncolog*))):ti,ab,kw (Word variations have been searched)	6113

#2	MeSH descriptor: [Rectal Neoplasms] explode all trees	2883

#3	#1 or #2	6113

#4	((KRAS or EGFR or APC or NTRK2 or BRAF or "B-raf" or "Kirsten rat sarcoma*" or "adenomatous polyposis coli" or "ptk receptor*" or "tyrosine kinase receptor*" or "epidermal growth factor receptor*" or "neurotrophic tyrosine receptor kinase*" or "transcriptomic signature*" or "RNA-based signature*" or "mismatch repair deficiency" or "microsatellite instability" or "high mutational burden*" or "genetic mutat*" or "cancer driver genes" or genomic* or "ErbB Receptor*")):ti,ab,kw (Word variations have been searched)	25830

#5	MeSH descriptor: [Proto-Oncogene Proteins B-raf] explode all trees	330

#6	MeSH descriptor: [Adenomatous Polyposis Coli] explode all trees	159

#7	MeSH descriptor: [ErbB Receptors] this term only	1021

#8	MeSH descriptor: [Receptor Protein-Tyrosine Kinases] explode all trees	3913

#9	MeSH descriptor: [Microsatellite Instability] this term only	108

#10	MeSH descriptor: [DNA Copy Number Variations] this term only	62

#11	(("copy number" Next (variant* or change* or polymorph*))):ti,ab,kw (Word variations have been searched)	79

#12	#4 or #5 or #6 or #7 or #8 or #9 or #10 or #11	27831

#13	((regress* or respon* or recur* or surviv* or "disease free" or metastas* or mortal* or death or (treat* Near/3 (outcome or effectiv* or efficac*)))):ti,ab,kw (Word variations have been searched)	856674

#14	MeSH descriptor: [Neoplasm Recurrence, Local] this term only	7580

#15	MeSH descriptor: [Neoplasm Regression, Spontaneous] this term only	11

#16	MeSH descriptor: [Survival] this term only	183

#17	MeSH descriptor: [Treatment Outcome] this term only	192820

#18	MeSH descriptor: [Disease-Free Survival] this term only	9760

#19	MeSH descriptor: [Neoplasm Metastasis] this term only	4593

#20	MeSH descriptor: [Mortality] this term only	1031

#21	MeSH descriptor: [Death] this term only	406

#22	#13 or #14 or #15 or #16 or #17 or #18 or #19 or #20 or #21	856674

#23	#3 and #12 and #22	207

**# Web of Science Search Strategy (v0.1)**

**# Database: Web of Science Core Collection**

\# Entitlements:

- WOS.IC: 1993 to 2024

- WOS.CCR: 1985 to 2024

- WOS.SCI: 1900 to 2024

- WOS.AHCI: 1975 to 2024

- WOS.BHCI: 2008 to 2024

- WOS.BSCI: 2008 to 2024

- WOS.ESCI: 2019 to 2024

- WOS.ISTP: 1990 to 2024

- WOS.SSCI: 1956 to 2024

- WOS.ISSHP: 1990 to 2024

\# Searches:

1: TS=(((Rect* or anus or anal) Near/2 (cancer* or tumo* or neoplasm* or malignan* or oncolog*)))	Date Run: Thu Jul 04 2024 11:57:09 GMT+0100 (British Summer Time)	Results: 55037

2: TS=((KRAS or EGFR or APC or NTRK2 or BRAF or "B-raf" or "Kirsten rat sarcoma*" or "adenomatous polyposis coli" or "ptk receptor*" or "tyrosine kinase receptor*" or "epidermal growth factor receptor*" or "neurotrophic tyrosine receptor kinase*" or "transcriptomic signature*" or "RNA-based signature*" or "mismatch repair deficiency" or "microsatellite instability" or "high mutational burden*" or "genetic mutat*" or "cancer driver genes" or genomic* or "ErbB Receptor*"))	Date Run: Thu Jul 04 2024 11:57:26 GMT+0100 (British Summer Time)	Results: 719610

3: TS=(("copy number" Near (variant* or change* or polymorph*)))	Date Run: Thu Jul 04 2024 11:57:53 GMT+0100 (British Summer Time)	Results: 13951

4: #3 OR #2	Date Run: Thu Jul 04 2024 11:58:03 GMT+0100 (British Summer Time)	Results: 727020

5: TS=((regress* or respon* or recur* or surviv* or "disease free" or metastas* or mortal* or death or (treat* Near/3 (outcome or effectiv* or efficac*))))	Date Run: Thu Jul 04 2024 11:58:47 GMT+0100 (British Summer Time)	Results: 13083610

6: #5 AND #4 AND #1	Date Run: Thu Jul 04 2024 11:59:00 GMT+0100 (British Summer Time)	Results: 1509

**Scopus**

( TITLE-ABS-KEY ( ( regress* OR respon* OR recur* OR surviv* OR "disease free" OR metastas* OR mortal* OR death OR ( treat* W/3 ( outcome OR effectiv* OR efficac* ) ) ) ) ) AND ( ( TITLE-ABS-KEY ( ( "copy number" W/1 ( variant* OR change* OR polymorph* ) ) ) ) OR ( TITLE-ABS-KEY ( ( kras OR egfr OR apc OR ntrk2 OR braf OR "B-raf" OR "Kirsten rat sarcoma*" OR "adenomatous polyposis coli" OR "ptk receptor*" OR "tyrosine kinase receptor*" OR "epidermal growth factor receptor*" OR "neurotrophic tyrosine receptor kinase*" OR "transcriptomic signature*" OR "RNA-based signature*" OR "mismatch repair deficiency" OR "microsatellite instability" OR "high mutational burden*" OR "genetic mutat*" OR "cancer driver genes" OR genomic* OR "ErbB Receptor*" ) ) ) ) AND ( TITLE-ABS-KEY ( ( ( rect* OR anus OR anal ) W/2 ( cancer* OR tumo* OR neoplasm* OR malignan* OR oncolog* ) ) ) )

**Ovid MEDLINE(R) and Epub Ahead of Print, In-Process, In-Data-Review & Other Non-Indexed Citations, Daily and Versions <1946 to March 20, 2026>**

1	((Rect* or anus or anal) adj2 (cancer* or tumo* or neoplasm* or malignan* or oncolog*)).ti,ab,kw,kf.	48492

2	exp Rectal Neoplasms/	58711

3	1 or 2	74788

4	(KRAS or EGFR or APC or NTRK2 or BRAF or "B-raf" or "Kirsten rat sarcoma*" or "adenomatous polyposis coli" or "ptk receptor*" or "tyrosine kinase receptor*" or "epidermal growth factor receptor*" or "neurotrophic tyrosine receptor kinase*" or "transcriptomic signature*" or "RNA-based signature*" or "mismatch repair deficiency" or "microsatellite instability" or "high mutational burden*" or "genetic mutat*" or "cancer driver genes" or genomic*).ti,ab,kw,kf.	667824

5	(("copy number" adj (variant* or change* or polymorph*)) or "ErbB Receptor*").ti,ab,kw,kf.	9981

6	exp Proto-Oncogene Proteins B-raf/ or exp Adenomatous Polyposis Coli/ or ErbB Receptors/ or exp Receptor Protein-Tyrosine Kinases/ or Microsatellite Instability/ or DNA Copy Number Variations/	222497

7	4 or 5 or 6	809413

8	(regress* or respon* or recur* or surviv* or "disease free" or metastas* or mortal* or death or (treat* adj3 (outcome or effectiv* or efficac*))).ti,ab,kw,kf.	9546539

9	neoplasm recurrence, local/ or neoplasm regression, spontaneous/ or Survival/ or treatment outcome/ or Disease-Free Survival/ or Neoplasm Metastasis/ or Mortality/ or Death/	1651955

10	8 or 9	10295059

11	3 and 7 and 10	1708

**Embase <1974 to 2026 March 19>**

1	((Rect* or anus or anal) adj2 (cancer* or tumo* or neoplasm* or malignan* or oncolog*)).ti,ab,kw,kf.	76642

2	exp rectum cancer/	71341

3	1 or 2	99177

4	(KRAS or EGFR or APC or NTRK2 or BRAF or "B-raf" or "Kirsten rat sarcoma*" or "adenomatous polyposis coli" or "ptk receptor*" or "tyrosine kinase receptor*" or "epidermal growth factor receptor*" or "neurotrophic tyrosine receptor kinase*" or "transcriptomic signature*" or "RNA-based signature*" or "mismatch repair deficiency" or "microsatellite instability" or "high mutational burden*" or "genetic mutat*" or "cancer driver genes" or genomic* or "ErbB Receptor*").ti,ab,kw,kf.	958473

5	("copy number" adj (variant* or change* or polymorph*)).ti,ab,kw,kf.	14494

6	K ras protein/ or epidermal growth factor receptor/ or EGFR signaling/ or APC protein/ or brain derived neurotrophic factor receptor/ or protein tyrosine kinase/ or mismatch repair/ or microsatellite instability/ or gene mutation/ or genomics/	933679

7	4 or 5 or 6	1594936

8	(regress* or respon* or recur* or surviv* or "disease free" or metastas* or mortal* or death or (treat* adj3 (outcome or effectiv* or efficac*))).ti,ab,kw,kf.	13005770

9	cancer regression/ or cancer recurrence/ or progression free survival/ or survival/ or disease free survival/ or metastasis free survival/ or metastasis/ or mortality/ or death/ or treatment outcome/	3401454

10	8 or 9	14002506

11	3 and 7 and 10	4583

**Search Name:	Iannish Sadien**

**Date Run:	23/03/2026 17:16:51**

**Comment:**

**ID	Search	Hits**

#1	(((Rect* or anus or anal) Near/2 (cancer* or tumo* or neoplasm* or malignan* or oncolog*))):ti,ab,kw (Word variations have been searched)	6734

#2	MeSH descriptor: [Rectal Neoplasms] explode all trees	3059

#3	#1 or #2	6734

#4	((KRAS or EGFR or APC or NTRK2 or BRAF or "B-raf" or "Kirsten rat sarcoma*" or "adenomatous polyposis coli" or "ptk receptor*" or "tyrosine kinase receptor*" or "epidermal growth factor receptor*" or "neurotrophic tyrosine receptor kinase*" or "transcriptomic signature*" or "RNA-based signature*" or "mismatch repair deficiency" or "microsatellite instability" or "high mutational burden*" or "genetic mutat*" or "cancer driver genes" or genomic* or "ErbB Receptor*")):ti,ab,kw (Word variations have been searched)	29182

#5	MeSH descriptor: [Proto-Oncogene Proteins B-raf] explode all trees	352

#6	MeSH descriptor: [Adenomatous Polyposis Coli] explode all trees	163

#7	MeSH descriptor: [ErbB Receptors] this term only	1078

#8	MeSH descriptor: [Receptor Protein-Tyrosine Kinases] explode all trees	2668

#9	MeSH descriptor: [Microsatellite Instability] this term only	122

#10	MeSH descriptor: [DNA Copy Number Variations] this term only	69

#11	(("copy number" Next (variant* or change* or polymorph*))):ti,ab,kw (Word variations have been searched)	99

#12	#4 or #5 or #6 or #7 or #8 or #9 or #10 or #11	30710

#13	((regress* or respon* or recur* or surviv* or "disease free" or metastas* or mortal* or death or (treat* Near/3 (outcome or effectiv* or efficac*)))):ti,ab,kw (Word variations have been searched)	922222

#14	MeSH descriptor: [Neoplasm Recurrence, Local] this term only	7713

#15	MeSH descriptor: [Neoplasm Regression, Spontaneous] this term only	11

#16	MeSH descriptor: [Survival] this term only	176

#17	MeSH descriptor: [Treatment Outcome] this term only	200369

#18	MeSH descriptor: [Disease-Free Survival] this term only	9520

#19	MeSH descriptor: [Neoplasm Metastasis] this term only	4845

#20	MeSH descriptor: [Mortality] this term only	991

#21	MeSH descriptor: [Death] this term only	414

#22	#13 or #14 or #15 or #16 or #17 or #18 or #19 or #20 or #21	922222

#23	#3 and #12 and #22	229

**# Web of Science Search Strategy (v0.1)**

**# Database: Web of Science Core Collection**

**# Entitlements:**

**- WOS.IC: 1993 to 2026**

**- WOS.CCR: 1985 to 2026**

**- WOS.SCI: 1900 to 2026**

**- WOS.AHCI: 1975 to 2026**

**- WOS.BHCI: 2008 to 2026**

**- WOS.BSCI: 2008 to 2026**

**- WOS.ESCI: 2021 to 2026**

**- WOS.ISTP: 1990 to 2026**

**- WOS.SSCI: 1956 to 2026**

**- WOS.ISSHP: 1990 to 2026**

**# Searches:**

1: TS=((Rect* or anus or anal) Near/2 (cancer* or tumo* or neoplasm* or malignan* or oncolog*))	Date Run: Mon Mar 23 2026 09:21:03 GMT+0000 (Greenwich Mean Time)	Results: 61318

2: TS=(KRAS or EGFR or APC or NTRK2 or BRAF or "B-raf" or "Kirsten rat sarcoma*" or "adenomatous polyposis coli" or "ptk receptor*" or "tyrosine kinase receptor*" or "epidermal growth factor receptor*" or "neurotrophic tyrosine receptor kinase*" or "transcriptomic signature*" or "RNA-based signature*" or "mismatch repair deficiency" or "microsatellite instability" or "high mutational burden*" or "genetic mutat*" or "cancer driver genes" or genomic* or "ErbB Receptor*")	Date Run: Mon Mar 23 2026 09:21:35 GMT+0000 (Greenwich Mean Time)	Results: 816055

3: TS=("copy number" Near (variant* or change* or polymorph*))	Date Run: Mon Mar 23 2026 09:22:09 GMT+0000 (Greenwich Mean Time)	Results: 15561

4: #3 OR #2	Date Run: Mon Mar 23 2026 09:22:15 GMT+0000 (Greenwich Mean Time)	Results: 824393

5: TS=(regress* or respon* or recur* or surviv* or "disease free" or metastas* or mortal* or death or (treat* Near/3 (outcome or effectiv* or efficac*)))	Date Run: Mon Mar 23 2026 09:22:47 GMT+0000 (Greenwich Mean Time)	Results: 14668355

6: #5 AND #4 AND #1	Date Run: Mon Mar 23 2026 09:22:54 GMT+0000 (Greenwich Mean Time)	Results: 1744

**Scopus**

( ( TITLE-ABS-KEY ( "copy number" W/1 ( variant* OR change* OR polymorph* ) ) ) OR ( TITLE-ABS-KEY ( kras OR egfr OR apc OR ntrk2 OR braf OR "B-raf" OR "Kirsten rat sarcoma*" OR "adenomatous polyposis coli" OR "ptk receptor*" OR "tyrosine kinase receptor*" OR "epidermal growth factor receptor*" OR "neurotrophic tyrosine receptor kinase*" OR "transcriptomic signature*" OR "RNA-based signature*" OR "mismatch repair deficiency" OR "microsatellite instability" OR "high mutational burden*" OR "genetic mutat*" OR "cancer driver genes" OR genomic* OR "ErbB Receptor*" ) ) ) AND ( TITLE-ABS-KEY ( ( rect* OR anus OR anal ) W/2 ( cancer* OR tumo* OR neoplasm* OR malignan* OR oncolog* ) ) ) AND ( TITLE-ABS-KEY ( regress* OR respon* OR recur* OR surviv* OR "disease free" OR metastas* OR mortal* OR death OR ( treat* W/3 ( outcome OR effectiv* OR efficac* ) ) ) )

**Results**

| Database | Results 04/07/2024 | Results 23/03/2026 |
| --- | --- | --- |
| Medline | 1499 | 209 |
| Embase | 3543 | 1040 |
| Cochrane Library | 207 | 22 |
| Web of Science | 1509 | 235 |
| Scopus | 2776 | 484 |
| After deduplication | 5616 | 1294 |

**References**

MCGOWAN, J., SAMPSON, M., SALZWEDEL, D. M., COGO, E., FOERSTER, V. & LEFEBVRE, C. 2016. PRESS peer review of electronic search strategies: 2015 guideline statement. *Journal of clinical epidemiology,* 75**,** 40-46.

RETHLEFSEN, M. L., KIRTLEY, S., WAFFENSCHMIDT, S., AYALA, A. P., MOHER, D., PAGE, M. J. & KOFFEL, J. B. 2021. PRISMA-S: an extension to the PRISMA statement for reporting literature searches in systematic reviews. *Systematic reviews,* 10**,** 1-19.

| **Authors, Year** | **Country** | **Study Design** | **Setting** | **Number**  **of Parti-cipants** | **Eligibility Criteria** | **Sequen-cing Method** | **Overall pCR rate (%)** | ***Pooled OS (%)*** | ***KRAS* Mutation Frequency (%)** | **Newcastle-Ottawa Score** | | |
| --- | --- | --- | --- | --- | --- | --- | --- | --- | --- | --- | --- | --- |
|  |  |  |  |  |  |  |  |  |  | **S** | **C** | **O/E** |
| Hammarstrom, 2024 | Sweden | Retrospective Cohort | Multicenter, regional | 552 | Invasive rectal adenocarcinoma <15cm from anal verge. | NGS | 15.2 | N/R | 44.2 | 3 | 2 | 3 |
| Chapman, 2023 | USA | Retrospective Cohort | Single, tertiary | 38 | First rectal adenocarcinoma (stage II/III) 4/2016-4/2020. | NGS | 18.4 | 96.2% (5 year) | 47.4 | 3 | 1 | 3 |
| Orlandi, 2023 | Italy | Retrospective Cohort | Single, tertiary | 39 | Surgery for stage II/III rectal adenocarcinoma (5/2011-6/2017). No metastases (CT). | NGS | 17.9 | 76.9% (5 year) | 35.9 | 4 | 0 | 3 |
| Chang, 2022 | Taiwan | Case-Control | Single, tertiary | 29 | Histologically confirmed and resected LARC with nCRT (2014-2018). | NGS | 31.0 | N/R | 48.3 | 2 | 1 | 3 |
| Zhou, 2021 | USA | Retrospective Cohort | Multicenter, national | 1886 | LARC with nCRT, age >18 years, KRAS data available (2010-2015) | NA | 10.3 | N/R | 36.1 | 3 | 1 | 3 |
| Toomey, 2020 | Ireland, USA | Retrospective Cohort | Two centers, tertiary & quaternary | 28 | LARC (<15cm anal verge), nCRT, and resection. | NGS | 17.9 | N/R | 32.1 | 3 | 1 | 3 |
| Bahnassy, 2020 | Egypt | Prospective Cohort | Single, tertiary | 70 | LARC: <15cm anal verge, resectable, histological diagnosis (3/2015-12/2015) | Sanger | 35.7 | 50.0% (5 year) | 34.3 | 4 | 1 | 3 |
| Otmani, 2020 | Morocco | Retrospective Cohort | Single, tertiary | 57 | Rectal adenocarcinoma 1/2012-10/2018. | Sanger | 31.6 | 91.2% (80-months) | 35.1 | 4 | 0 | 3 |
| Greenbaum, 2019 | USA | Retrospective Cohort | Single, teriary | 21 | LARC with surgical resection after nCRT. | NGS | 38.1 | N/R | 14.3 | 4 | 1 | 2 |
| Jiang, 2019 | China | Retrospective Cohort | Single, tertiary | 74 | Stage II/III rectal adenocarcinoma, nCRT, resection, 1/2013-6/2016. | NGS | 17.6 | N/R | 35.1 | 3 | 1 | 3 |
| Chow, 2016 | USA | Retrospective Cohort | Multicenter, national | 229 | LARC (Stage II/III, <15cm anal verge), no metastases (CT). | Sanger & NGS | 25.7 | N/R | 41.9 | 4 | 1 | 3 |
| Martellucci, 2015 | Italy | Prospective Cohort | Single, tertiary | 76 | LARC (2007-2012) | Sanger | 9.2 | 96.1% (33-months) | 34.2 | 4 | 0 | 3 |
| Russo, 2014 | USA | Retrospective Cohort | Single, quaternary | 47 | LARC with preoperative CRT (7/2005-6/2010). | Capillary electrophoresis | 36.1 | N/R | 42.6 | 4 | 1 | 3 |
| Davies, 2011 | USA | Retrospective Cohort | Single, tertiary | 67 | Rectal adenocarcinoma treated with nCRT and sufficient tissue. | NGS | 16.4 | 58.6% (54 months) | 35.8 | 3 | 1 | 3 |
| Bengala, 2010 | Italy | Retrospective Cohort | Single, tertiary | 141 | LARC with preoperative chemotherapy (5/1998-10/2005). | Sanger | 14.8 | 71.5% (5 year) | 19.1 | 3 | 1 | 3 |

**Table S1:**Characteristics of included studies. USA: United States of America; OS: Overall Survival, S: Selection, C: Comparability, O/E: Outcomes (Cohort)/Exposures (Case-Control); LARC: Locally Advanced Rectal Cancer; CT: Computer Tomography scan; (n)CRT: Neoadjuvent Chemoradiotherapy; NGS: Next Generation Sequencing. N/R: Not Reported. *All studies assayed all KRAS exons with the exception of Zhou et al. 2021 (not reported); Bahnassy 2020 and Bengala 2010 (Exon 2 only); Davies 2011 (Exon 2 and Codon Q61; Chow 2016 (Exon 2 & 3 only).*

**Figure S1:** Heterogeneity analysis. **a.** Funnel plot of the 15 included studies showing relationship between reported odds ratio and standard error. **b.** Scatter plot of influence of individual studies on pooled result and heterogeneity contribution.

**Figure S2:** Influence analysis metrics.

**Figure S3:** Forest plot of odds ratios after exclusion of influential study by Zhou et al.

| **Characteristic** | **N** | ***KRAS* wildtype**  N= 228 | **Exon 2 mutated**  N=130 | **Non-exon 2 mutated**  N=32 | **Adjusted *P* value** |
| --- | --- | --- | --- | --- | --- |
| **Sex**  Female  Male | 398 | 81 (36%)  147 (64%) | 68 (52%)  62 (48%) | 12 (38%)  20 (63%) | 0.059 |
| **Tumour size (cm)** | 375 | 4.3 (3.5-5.6) | 4.5 (3.7-5.6) | 4.0 (3.2-5.0) | 0.6 |
| **Rectal location**  Lower  Middle  Upper | 395 | 52 (23%)  104 (46%)  71 (31%) | 38 (29%)  53 (41%)  38 (29%) | 9 (29%)  16 (52%)  6 (19%) | 0.8 |
| **BMI** | 140 | 27 (23-31) | 27 (23-31) | 28 (26-32) | 0.8 |
| **cT stage**  T1  T2  T3  T4 | 287 | 0 (0%)  5 (3.1%)  141 (87%)  16 (9.9%) | 1 (1.1%)  10 (11%)  69 (73%)  15 (16%) | 0 (0%)  1 (4.2%)  22 (92%)  1 (4.2%) | 0.2 |
| **cN stage**  N0  N+ | 380 | 42 (19%)  175 (81%) | 26 (20%)  101 (80%) | 8 (28%)  21 (72%) | 0.8 |
| **cM stage**  M0  M1 | 396 | 228 (100%)  0 (0%) | 130 (100%)  0 (0%) | 32 (100%)  0 (0%) |  |
| **Neoadjuvant**  Chemo only  Consolidation  Induction  CRT only | 391 | 8 (3.6%)  61 (27%)  36 (16%)  120 (53%) | 3 (2.3%)  33 (26%)  23 (18%)  69 (54%) | 2 (6.3%)  7 (22%)  7 (22%)  16 (50%) | 0.9 |
| **Adjuvant chemo**  No  Yes | 226 | 94 (74%)  33 (26%) | 60 (73%)  22 (27%) | 14 (82%)  3 (18%) | 0.8 |

**Table S2:** Baseline characteristics for patients in survival analysis. *P* value adjusted for multiplicity testing.

**Figure S4:** Transcriptomic analysis and cell type deconvolution. **a.** Volcano plot showing differential gene expression analysis comparing non-exon 2 *KRAS* mutant samples versus exon 2 mutants. **b-d.** Box plots showing CIBERSORT cell type deconvolution scores for CD8+ T cells, regulatory T cells and activated NK cells. Wilcoxon test.
